# Supplementary material for: Are carbon emissions trading and green financial instruments synergistic? -Comprehensive quantitative research based on content analysis
Source: PLoS One. 2024 Mar 7;19(3):e0298601. doi: 10.1371/journal.pone.0298601 (PMC10919601; doi:10.1371/journal.pone.0298601)
Supplement: S1 Data — (ZIP) [file pone.0298601.s001.zip › Data/地级市Z_F工作报告.zip!/║╙─╧/─╧╤⌠╩╨/└┤╫╘╣1⁄2╓┌║┼í╛┬φ┐╦╩2╛▌═°í┐_2007.docx]

工作回顾

--综合实力显著提升。全市生产总值完成1201亿元,比上年增长13.5%。固定资产投资完成514.4亿元,增长36.3%,三年累计1195亿元,"双千亿"目标超额完成。地方财政一般预算收入36.3亿元,增长25.9%;支出109.1亿元,增长41.9%。全社会消费品零售总额392.5亿元,增长15.6%。城乡居民储蓄存款余额532.9亿元,增长13.8%。城镇居民人均可支配收入8913元,农民人均纯收入3386元,分别增长13.8%、17%。

--工业经济快速发展。全市工业增加值完成546.6亿元,增长16.7%。限额以上工业增加值、销售收入、利税分别增长22.9%、38.1%、52.2%,综合效益指数达到210%、提高37个百分点。4家企业入选"全国创新能力行业十强",6家企业跻身全省工业百强,10家企业荣获全省百家优秀民营企业称号,2个产品被评为"中国名牌"。工业园区建设步伐加快,成为工业发展的重要引擎。光电、电力、生物质能等产业开始兴起,成为南阳工业新亮点。鸭电二期、热电一期、迅天宇多晶硅、中光学集团光学引擎、全国首条1000千伏特高压开关站等重点项目开工建设。天池抽水蓄能电站和孟山(高庄)核电项目前期工作取得新进展。

--"三农"工作全面加强。认真落实各项支农惠农政策,扎实推进新农村建设,农业结构不断优化,农民收入明显提高,农村生产生活条件逐步改善。粮食总产527.2万吨,创历史最高水平。大宗粮食作物优质率达85%以上,新认定无公害农产品生产基地37万亩、达到517万亩。植树造林116.5万亩,提前实现全市平原绿化高级达标。新增有效灌溉面积15.1万亩、节水灌溉面积17.2万亩、旱涝保收田11.3万亩,完成小流域治理186.6平方公里,实现全省"红旗渠精神杯"八连冠。解决农村16.3万人安全饮水。新发展农村沼气5.1万户。新建改建农村公路4664公里,新增1300个村通水泥(油)路。新农村建设全面启动,试点村建设初见成效。

--城乡面貌明显改善。南阳中心城市相继荣获"中国优秀旅游城市"、"国家园林城市"称号。城市总体规划和市域城镇体系规划顺利完成,城镇建设迈出新步伐,全市城镇化率提高2个百分点、达到32%。旅游基础设施建设力度加大,旅游景区开发品位提升,南阳伏牛山被评为世界地质公园,内乡县衙、西峡鹳河漂流被命名为国家4A级景区。交通建设加速推进,信南高速公路建成通车,宁西铁路客运开通。蒲山矿区、白河流域环境综合整治取得明显成效。各县市区在工业、城建等方面迈出新步伐,县域经济呈现明争暗赛、竞相发展的良好局面。

--改革开放步伐加快。国有企业改革稳步推进。企业上市实现零的突破,新纺公司成功上市,陆德筑机在全省率先实现民营股权上市。分配制度改革有序展开,公务员登记和机关事业单位人员工资套改基本完成,企业离退休人员养老金如期调整。农村综合改革深入推进,财税、粮食流通、供销系统等领域的改革取得新进展。以"对外开放推动年"活动为载体,全面扩大对外开放。全年引进市外资金105亿元。实际利用外资6704万美元,增长39.4%。外贸出口3.4亿美元,增长44%。

--和谐社会建设扎实推进。"十件实事"全部落实。就业再就业工作得到加强,城镇登记失业率控制在3.4%以内。社会保障体系不断完善,农村居民最低生活保障制度初步建立,城镇居民最低生活保障标准全面提高。科教兴宛战略深入推进,荣获"全国科技进步先进市"。教育、文化、体育事业稳步发展。公共卫生服务体系进一步健全,被评为全国首个"农村中医工作先进市"。广播电视工作成效显著,数字电视整体平移工作进入全国先进行列。社会主义荣辱观教育和精神文明创建活动不断深入,民主法制建设进一步加强。社会治安防控体系逐步完善,信访秩序和安全生产形势持续好转,社会大局保持稳定。荣获全省"综治先进市"、"信访工作先进市"、"安全生产优秀市"、"食品安全工作先进市"称号。人口自然增长率控制在5.05‰,荣获"全国奖扶工作先进市"称号,被确定为"全国人口计生工作综合改革试点市"。工商、税务、物价、金融、审计、统计、民政、国土、环保、房产、邮政、通信、质量监督、食品药品监督、检验检疫、海关、电力、烟草、盐业、人防、地震、气象、史志、民族宗教、民兵预备役和双拥共建等工作都取得了新的成绩。

各位代表,过去一年,我市主要发展目标超出预期,大事、喜事多,亮点纷呈,经济社会发展迈上新台阶。这些成就的取得,是省委、省政府和市委正确领导的结果,是全市人民共同奋斗的结果,是人大、政协等各方面监督支持的结果。在此,我代表市人民政府,向全市人民,向各位人大代表、政协委员,向驻宛部队、武警官兵和政法干警,向中央、省驻宛单位,向各民主党派、工商联、人民团体和各界人士,向所有关心支持南阳发展的国内外朋友,表示崇高的敬意和衷心的感谢!

在充分肯定去年工作的同时,我们也清醒地看到面临的巨大压力和严峻挑战。一是随着国家加强和改善宏观调控,项目、市场准入门槛提高,土地、环境、资源约束趋紧,对我们调整经济结构和转变增长方式提出了新的更高的要求。二是区域竞争日趋激烈,周边地市加速发展的势头咄咄逼人,前面标兵跑得更快、后面追兵追得更紧,我们面临不进则退、慢进亦退的严峻挑战。三是我市城乡建设历史欠帐较多,一些领域改革相对滞后,投资环境有待改善,政府自身建设也存在一些问题。对此,我们一定要保持清醒的头脑,在今后的工作中努力加以解决。

折叠编辑本段目标任务

2007年是我市全面落实科学发展观、构建和谐社会的重要一年。综观全局,尽管我们面临不少挑战,但更面临诸多难得机遇和有利条件。从宏观形势看,国内外投资向中西部转移步伐加快,有利于我们大规模承接产业转移;国家促进中部崛起和新农村建设等政策陆续出台,省里大力推进"两个跨越"和"两个建设",并把南阳列入全省"第二方阵"、确定为全省光电等新型工业化基地,为我们提供了重大政策机遇。从发展基础看,南阳自然、人文资源丰富,交通区位优势日益凸显,农业基础较好,工业发展势头强劲,重大项目进展顺利,后发优势明显,发展潜力巨大,一个富有生机、充满希望的南阳正展现在世人面前。从发展动力看,市四次党代会勾画了建设富强美好和谐新南阳的宏伟蓝图,极大地鼓舞了广大干部群众干事创业的热情,加快发展、跨越前进的愿望更加强烈,精诚团结、心齐劲足的局面已经形成。只要全市上下抓住机遇,乘势而上,奋力拼搏,南阳经济社会发展就一定能够不断迈上新台阶,在中原崛起中发挥重要作用。

今年政府工作的总体要求是:以邓小平理论和"三个代表"重要思想为指导,认真贯彻落实市四次党代会和市委经济工作会议精神,坚持以科学发展观统领经济社会发展全局,围绕建设富强美好和谐新南阳目标,积极实施工业强市、开放带动和创新推动战略,强力推进传统农区工业化、中心城市建设、新农村建设、文化旅游产业发展四大突破,致力改善民生,加快构建和谐社会,促进经济社会又好又快发展。

今年经济社会发展的主要预期目标是:生产总值增长13%;一般预算收入增长15%;全社会固定资产投资增长20%以上;社会消费品零售总额增长13%;外贸出口增长15%,实际利用外资增长20%;万元生产总值能耗降低4.5%左右;化学需氧量、二氧化硫排放量分别减少4.5%和4.2%;城镇居民人均可支配收入增长8%,农民人均纯收入增长6%;居民消费价格涨幅控制在3%左右;城镇登记失业率控制在4%以内;人口自然增长率控制在6.5‰以内。

根据上述总体要求和奋斗目标,今年重点抓好以下工作:

(一)实施工业强市战略,推进传统农区工业化新突破。南阳工业正进入一个大发展、大提升、大跨越的关键期。坚持工业主导地位不动摇,大力实施工业强市战略,强力推进工业经济更大规模、更高层次的发展,尽快把南阳建设成为全省重要的新型工业化基地。

突出抓好重点项目。积极开展"项目推进年"活动,把46个投资超亿元、总投资186.6亿元、今明两年开工或竣工的重点项目,作为我市工业强市战略的"发动机计划"项趴,确保今年完成工业投资210亿元,其中重点工业项目完成投资110亿元。天冠集团30万吨玉米综合深加工及3万吨可降解塑料、防爆集团大型高效矿用防爆主通风机、安棚碱矿三期年产100万吨纯碱、二胶厂年产1600万平方米CTP数码印刷版材、红宇机械厂年产1万台冷藏专用运输车等24个计划新开工项目,今年要按期或尽早开工。迅天宇多晶硅一期工程、金光数显年产10万台MD数字高清大屏幕电视机、新纺10万锭环锭纺、中光学年产24万台DLP和10万台LCOS光学引擎等18个项目,年内要按期竣工。其他28个项目,确保完成年度投资计划,其中南阳热电、鸭电二期、方城风电等11个项目力争年内部分投产。同时,扎实做好天冠72万吨蛋白饲料、抽水蓄能电站、孟山核电、宛西电厂等重大项目前期工作。

积极培育大型骨干企业。不分性质、属地和隶属关系,筛选确定河南油田、天冠、南纺、新纺、防爆、二胶、中光学、宛西制药、西保、龙成、淅铝、安棚碱矿等20家实力强、潜力大、前景好的骨干工业企业予以重点扶持,尽快培育一批主业突出、行业领先、有自主知识产权、核心竞争力强的大公司大集团。力争河南油田年销售收入突破120亿元,龙成达到40亿元,天冠、淅铝超过30亿元,西保、新纺、鸭电达到20亿元,其他超10亿元的企业达到12家。同时,注重扶持中小企业特别是优质成长型企业发展,健全担保体系和激励机制,促进中小企业做大做强,培育一大批年产值超亿元、5亿元的行业"小巨人"。从今年开始,在全市工业企业中开展"十大纳税企业"、"十大高成长型企业"评选活动和争创中国名牌、驰名商标活动,市财政拿出专款予以奖励。

发展壮大优势产业。依托八大优势产业,围绕拉长产业链条,开发下游、高端产品,大力发展配套产品,实行规划引导、政策驱动,强化保证措施,推进工业集约化、园区化和基地化发展,形成一批集中度高、关联性强的产业集群。集中培育、致力打造光电、电力、生物能源、纺织四大全省重要的产业基地,大力发展、改造提升医药、机电、油碱化工、冶金建材、汽车配件等传统优势产业。积极支持光学引擎、多晶硅、纤维乙醇、生物柴油、全降解塑料、新型能源、超硬材料及制品、大型石油钻采设备、生物制药等高新技术产业化项目建设,尽快形成规模优势。加强企业研发中心建设,推动关键技术和重大装备研发,纳入省百户重点企业、50家重点高成长型企业和市定20家重点企业,争取今年全部建立市级以上技术中心,构建自主创新平台。

推进工业园区建设。把工业园区作为工业发展的主战场,完善园区功能,理顺管理体制,创新推进机制,高起点规划、高标准建设、高水平管理、高速度推进,全面提高园区发展水平。今年高新区、生态工业园区、龙升工业园区新引进超亿元项目分别达到5个以上,高新区工业销售收入和高新技术产品销售收入分别突破50亿、20亿元。加快官庄、鸭河工业区规划建设步伐,支持各县市特色工业园区和产业集聚区发展,推动全市工业规模膨胀、质量提升,努力把工业园区建成新型工业化的先导区、功能齐全的城市新组团。

(二)加大"三农"投入力度,推进新农村建设新突破。农业、农村、农民问题,是关系全面建设小康社会的重大问题。要千方百计增加"三农"投入,以加快发展现代农业、增加农民收入和强化农村公共服务为重点,扎实推进社会主义新农村建设。

大力发展现代农业。按照优质、高产、高效、生态、安全的要求,推进农业区域化布局和专业化、标准化、品牌化生产。重点抓好600万亩优质小麦、200万亩专用玉米、50万亩优质水稻、120万亩脱毒红薯、130万亩优质棉花、200万亩油料、100万亩蔬菜、100万亩小辣椒、20万亩烟叶、200万亩中药材生产基地建设。集中支持畜牧业重点县市和畜牧业优势区域发展,新建畜禽养殖小区80个以上,健全县级动物防疫体系。完成造林39.2万亩和省下达的林业生态县创建任务;积极发展以速生丰产林为主的工业原料林,推进林纸一体化。切实抓好鸭河口灌区10万亩和卧龙、社旗、方城等现代农业示范区建设。大力发展农副产品加工业和农民专业合作组织,提高农业产业化水平。筛选确定73家龙头企业和10家规模较大的农副产品批发交易市场,予以重点扶持,力争新增10家年销售收入超亿元的龙头企业,新增5个年交易额5000万元以上的农产品交易市场。

加快农村基础设施建设和社会事业发展。切实把基础设施建设和社会事业发展的重点转向农村,财政新增教育、卫生、文化等事业经费主要用于农村,逐步加大土地出让收益用于农村的比重,全面落实各项支农惠农政策。今年市本级用于"三农"的资金新增加5000万元以上。加强农田水利基本建设,新增有效灌溉面积12万亩、旱涝保收田和节水灌溉面积各10万亩;力争3年内完成12座中型、79座小一型病险水库除险加固任务。加大农业综合开发、扶贫开发和移民工作力度,确保完成20万亩中低产田改造任务,解决和巩固7万农村贫困人口温饱问题。加快农村道路、沼气、饮水、供电、通信等基础设施建设,改善农民生产生活条件。完成3696公里"村村通"工程建设任务,实现全市所有行政村通水泥(油)路,搞好道路维修养护;解决20万人安全饮水问题;新增农村沼气用户7万户;新增无电村通电户数14万户。大力发展农村社会事业。全部免除农村义务教育阶段学杂费,继续对农村贫困家庭学生免费提供教科书并补助寄宿生生活费;搞好农村1865所中小学现代远程教育工程建设,完成500所农村中小学校舍维修改造任务。新增有线电视和无线数字电视用户4万户,完成904个20户以上自然村通广播电视任务。建设60个乡镇综合文化站和100个村级文化大院。完成83个乡镇卫生院改、扩建和11个县市"120"急救指挥中心建设任务,13个县市区全部推行新型农村合作医疗制度。

扎实推进新农村规划建设。完成全市382个市县两级试点村、中心城区近郊200个行政村和全市30%非试点村的整体规划。各项涉农资金及项目向试点村倾斜,加强试点村环境整治,重点解决村内道路、给排水设施、垃圾处理、文化娱乐设施等突出问题。深入开展市直部门帮建活动,利用两年时间全面完成市级129个试点村的包建任务。借鉴扶贫开发"整村推进"的经验,加大城市近郊和其他非试点村规划建设力度。结合新农村建设,完成300个村级组织活动场所建设任务。

(三)围绕加快城镇化进程,推进中心城市建设新突破。实施中心城市带动战略,促进县城和小城镇协调发展,大力发展房地产业,构建现代交通体系,加快城镇化进程,力争全市城镇化率提高2个百分点。

深入开展"四城联创",加快中心城市发展步伐。充分认识"四城联创"的重大意义,把"四城联创"作为中心城市发展的重要载体,鼓劲加压,强力推进,巩固扩大国家园林城、优秀旅游城创建成果,加大国家卫生城、文明城创建力度,加快向豫鄂陕区域性特大城市和生态宜居城市的目标迈进。坚持新区扩张与旧城改造并举,突出"一带三区"(白河两岸生态景观带和东北分区、生态工业园区、龙升工业园区),实行规划、建设、经营、管理四位一体,提升城市品位,加速做大做强中心城市。一是搞好城市规划。认真编制并严格执行城市分区规划、专业规划和控制性详规,充分发挥规划的龙头作用。对白河两岸等重点地段的规划建设实行严格控制。对新建小区、城中村改造的居住小区,在学校、社区服务、市场建设及供热、供气、绿地建设等公共基础设施方面执行强制配套标准,确保新建、改建小区都成为宜居小区。继续实行建筑方案优选制度,加强规划实施的动态管理。加大规划执法力度,依法拆除违章建筑。严格规划控制,维护规划的严肃性和权威性。二是加快城市建设。抓好白河大桥、第二橡胶坝、迎宾大道及北京大道南延配套工程建设,确保白河大桥8月底前竣工通车,第二橡胶坝扩建工程6月20日前完工。集中搞好"四城联创"第二批重点工程。对七一路、文化路、梅溪路、建设路、新华路等5条主干道和6个城区出入市口进行综合整治。新修车站北路、张衡东路,高标准改造工农路中心医院门前段和仲景路全线。搞好净土庵、东关、溧河店、王营、东华等5个"城中村"改造。新建一批垃圾中转点和城市水冲厕所,增设一批垃圾箱。投入500万元,抓好一批城区支路、背街小巷路面、污水管线和路灯改造。利用2-3年时间,将全市支路、背街小巷道路全部改造一遍。启动东北分区建设。搞好白河城区段综合整治。扎实做好城市内河综合整治前期工作,积极创造条件,逐步把6条城市内河建设成为南阳新的生态景观带。搞好"一环一带四片六线"("一环"即环城高速绿化带,"一带"即白河生态绿化带,"四片"即独山、兰营水库、黄台岗、二十里岗四个片林,"六线"即六大出入市口两侧通道绿化)造林绿化和管护工作,构建中心城市生态绿化体系。加强城市社区建设,提高社区服务水平,优化社区环境,创建社区文化,维护社区稳定,推进居民自治,建设和谐社区。三是拓展城市经营。用开放的理念、市场的办法搞城建,走以城养城、以城建城之路。建立完善城市土地收购、储备、出让机制,全面清理闲置土地。严格执行经营性建设用地先详规再"招拍挂"制度,搞活土地资产经营。放开城市经营性基础设施建设和管理项目,把一批有效益、有回报的经营性公共项目推向市场。四是强化城市管理。理顺城市环卫管理体制,着力解决"看得见的人管不住、管得住的人看不见"的问题。对城市环卫清扫保洁,推行"划定区段、招标承包、竞争上岗、绩效挂钩、奖优罚劣"的管理模式,落实门前"三包"制度,提高清扫保洁质量。调整充实市城建监察支队,成立区级城建监察大队,形成"三级管理、两级执法"的城管执法体制。整治占道经营、店外经营和流动摊贩,规范整顿停车场。坚持公交优先,优化线路布局,增加公交车辆班次,取缔非法营运,搞好交通管理,积极化解交通拥堵问题。

加强县城和小城镇建设。积极支持11个县市搞好城市规划建设管理,创建"园林城市",提高城市品位,加快发展步伐。鼓励基础条件好、产业支撑强、辐射半径大的县(市)城,发展成为中等城市。大力发展专业镇和沿线、沿边城镇带,继续开展争创星级镇活动,加大扶持力度,突出抓好50个重点镇建设,支持石佛寺、穰东等一批重点城镇发展成为小城市,形成优势互补、各具特色的南阳中小城市格局。

大力发展房地产业。重点发展面向广大群众的普通商品住房,扩大普通商品房供应,加快经济适用房和廉租住房建设。制订优惠政策,鼓励房地产企业实施连片开发和规模化运作,引导房地产企业参与城中村和旧城改造。支持房地产开发企业通过兼并、收购和重组等形式做大做强,培育一批大型房地产企业集团。全面放开房地产市场,积极吸引国内知名房地产企业来宛投资开发,借助外力加速提升南阳城市品位和档次。整顿、规范房地产市场秩序,促进房地产业健康、有序、快速发展。

构建现代综合交通体系。积极支持焦柳铁路电气化改造。加快宛坪、岭南、驿宛3条高速公路建设,确保宛坪高速11月底前通车,驿宛高速和岭南高速南召县城以南55公里路段年底前通车,新增高速公路通车里程235公里,总里程突破500公里,80公里环城高速全线贯通。完成投资6亿元、561公里的干线公路建设任务,搞好道路养护管理和绿化美化。扎实做好邓内高速、三门峡至淅川、武陟至西峡高速、中心城区4条环城高速引线、312国道通向桐柏碱化工城、南阳油田和社旗县城分别至中心城区的快速通道等工程的前期工作。

(四)围绕建设文化旅游强市,推进文化旅游产业发展新突破。发挥南阳文化底蕴丰厚、生态优良的优势,坚持文化旅游两篇文章一起做,加快文化旅游资源大市向文化旅游强市跨越。

繁荣发展文化事业。编制文化事业发展规划,制定南阳国家历史文化名城保护办法,加大政府对公益性文化事业投入力度。筹建市综合博物馆,加强县级文化馆、图书馆、乡镇文化站和新农村书屋等公共文化服务体系建设。实施文化信息资源共享和农村电影放映工程。繁荣发展新闻出版、广播影视、文学艺术,培育文化精品。搞好全国第五次文物普查和重点文物、珍贵档案、非物质文化遗产的保护、开发及利用。深入开展"扫黄打非",加强文化市场管理。

加快发展文化产业。围绕打响"玉雕之都"品牌,整合南阳玉雕资源,鼓励支持玉雕产品标准化生产、大师化创作、品牌化经营,促进玉雕产业上档升级。依托张仲景医药文化品牌,坚持产、加、销、医、教、研"六位一体",发展壮大中医药文化产业。加快全市有线电视网络整合和数字化改造。积极培育出版、广告、会展、文化创意、动漫制作等现代文化产业和骨干企业。加强文化产业项目库建设,搞好文化项目推介和招商工作。把文化产业列入政府目标管理和国民经济统计体系,研究修订南阳文化产业发展规划和配套政策,明确目标,分解任务,快速推进。深化文化体制改革,认真落实文化体制改革方案和配套政策,加大经营性文化事业单位改革力度。积极扶持民营文化企业健康发展。

加速壮大旅游产业。抓住省里支持伏牛山生态旅游开发的机遇,充分发挥南阳伏牛山世界地质公园、中国优秀旅游城市和国家历史文化名城的品牌优势,聚焦亮点、整合资源、提升水平,集中精力把"宝天曼"打造成为国内一流、国际知名的精品景区,带动南阳旅游产业加速发展壮大。加大旅游基础设施建设力度,加快伏牛山等景区旅游快速通道建设,争取"十一"黄金周前建成宝天曼至真武顶之间的旅游通道;搞好旅游道路两侧绿化、美化,控制公路两侧用地,严禁沿路乱搭乱建,拆除违章建筑,整治环境卫生,逐步把每条旅游线路都建成生态路、景观路。深化旅游体制改革,按照"政府主导、科学规划、多元投资、市场运作"的模式,经政府批准对一些具备条件的景区转让开发权和经营权,鼓励大企业、大集团参与旅游资源开发经营,提高景区建设的档次和品位。积极推进旅游精品景区建设,建立创A激励机制,引导景区完善硬件、提升软件、增强功能、丰富内涵,新创2A级以上景区(点)9个。加大景区和旅行社资源整合力度,培育旅游骨干企业集团和旅游品牌。规范整顿旅游市场秩序,加强对A级景区、星级饭店和旅行社的动态管理,提高旅游接待服务水平。加大旅游包装、宣传促销力度,全力扩大客源市场,力争全年接待游客850万人次,旅游综合收入达到45亿元。

积极发展各类服务业。以文化旅游产业为龙头,带动各类服务业全面发展。编制全市服务业发展总体规划和专项规划,出台政策,重点发展物流配送、金融保险、科技服务、商务服务等生产性服务业,着力培育信息、咨询、娱乐、中介等新兴服务业,改造提升餐饮、运输、商贸等传统服务业。抓住服务业全面开放的机遇,吸引国内外知名生产性服务企业集团来宛设立研发中心、运营中心,积极争取和支持国内外各类金融单位来宛设立分支机构和开展相关业务,鼓励支持境外资本和民营资本在更广泛的领域参与服务业发展。研究出台现代服务业产业导向目录和促进现代服务业发展的优惠措施,落实服务业发展引导资金,推动服务业加速发展。

(五)强力推进改革开放,增强经济发展动力。积极实施开放带动和创新推动战略,加大改革攻坚力度,创新发展模式,提高对外开放水平,不断增强经济社会发展的动力和活力。

全面深化各项改革。加快国有集体企业改革步伐。坚持分类指导,一企一策,全面推进,确保28家市属国有工业企业今年全部完成改制任务,集体所有制企业改制取得明显进展。加大南阳商场、百货站等有较大规模和影响的流通企业改制力度,务求实现突破。改革投融资体制,拓宽融资渠道,通过资产重组、资本运作,推动企业重组和产业整合。高度重视企业上市工作,争取利达光电公司成功实现上市,中光学、防爆等6家企业成为上市后备企业。扎实推进农村综合改革。深化和完善乡镇机构改革,加快农村义务教育改革和县乡财政管理体制改革,逐步建立精干高效的农村行政管理体制、政府保障的农村义务教育体制、覆盖城乡的公共财政体制。稳步推进收入分配制度改革。认真组织实施公务员工资和事业单位收入分配制度改革,确保离退休人员基本养老金按时足额发放。同时,深入推进物价、交通、科技、财政、公用事业等领域的改革。

发展壮大非公有制经济。把非公有制经济作为最具爆发力的增长点,与国有经济一视同仁,鼓励、支持、引导非公有制经济加速发展壮大。进一步完善、落实促进非公有制经济发展的政策措施,鼓励非公有制经济参与国有企业改革,进入基础设施、公用事业、金融服务和社会事业等领域;鼓励金融资金和各类人才向非公有制经济流动;鼓励非公有制经济推进机制、技术和管理创新,向科技化、外向型、集团化方向发展,集中培育一批非公有制企业集团;鼓励支持非公有制业界人士参政议政,重奖对社会贡献大的企业家;加强对非公有制经济的引导和管理,改进服务,保护非公有制经济的合法权益,在全社会营造创业者光荣、纳税人至上的浓厚氛围。

创新发展模式。研究出台增强自主创新能力、建设创新型南阳的规划及政策措施,深化全国科技进步先进市创建工作。实施国家、省、市科技计划,抓好国家级高新科技创业园、省级光电产业园区和一批重点实验室、工程技术中心、博士后科研工作站、科技孵化器建设,培育一批高新技术企业群体。实施"人才强市"战略,加大对高层次人才引进力度,积极筹建南阳市高层次人才住宅,建立一支在本行业、本领域具有较强自主创新能力的高层次人才队伍。建立节能降耗目标责任制,完善相关统计监测、考核指标体系,着力抓好冶金、建材、钢铁、火电、化工等重点行业和企业的节能降耗工作。搞好秸秆纤维乙醇、生物柴油技术研发等试点示范项目建设和循环经济试点工作。加强南水北调中线工程水源、中心城区饮用水源和淮源"三源"保护,确保饮水安全。加大环境污染防治力度。集中力量对白河、唐河、中心城区内河等重点流域水污染进行综合整治。加快市及县城垃圾处理场和新野、邓州、淅川、唐河、社旗污水处理厂建设步伐,确保年内全部建成并正常运营。搞好医药、化工、水泥、电力等重点污染行业的监控和综合整治,淘汰落后生产工艺,削减排污总量。强化执法监督管理,认真落实环保目标责任制,对各县市区的环境保护实行一票否决。继续实施退耕还林、天然林保护和城镇生态建设工程,进一步改善生态环境。坚决实行最严格的土地管理制度,认真执行土地利用总体规划和年度计划,新增建设用地主要用于保障重点项目建设需要。深入开展"三项整治",挖掘、盘活存量建设用地。建立健全主要矿藏有偿使用和生态补偿机制,治理整顿矿业秩序,禁止乱采乱挖。加大地质勘查投入,积极寻求战略合作伙伴,尽快将我市的矿产资源优势转化为经济优势。

全面扩大对外开放。开放的力度有多大,发展的空间就有多大。进一步解放思想,把开放带动作为经济发展的主战略,全方位、宽领域、深层次扩大对外开放,以大开放促进大发展。创新举办节会活动,完善落实招商引资激励政策,进一步拓宽招商领域,改进招商方法,创新招商方式,建好招商队伍,强化目标责任,注重招商实效,努力实现招商引资大突破。力争全年实际利用外商直接投资8000万美元,引进市外资金130亿元,引进投资超亿元项目50个。加强市外经济技术合作与交流,鼓励各类企业走出南阳参与国内外市场竞争。积极发展对外项目工程承包,扩大境外劳务输出规模。优化出口产品结构,重点培育机电、特色农产品等一批优势出口商品,力争全年出口额超千万美元的企业达到15家以上,超百万美元的企业达到35家以上。

(六)以解决民生问题和促进社会发展为重点,致力构建和谐南阳。把关注民生、重视民生、保障民生、改善民生放在全市工作重中之重的位置,把共同建设、共同享有贯穿于和谐社会建设的全过程,认真办好十件实事,全面落实各项惠民政策,加快发展社会事业,加强精神文明和民主法制建设,努力促进社会和谐发展。

扎实做好就业和社会保障工作。继续实施促进就业的财政、税收和金融政策,积极支持自主创业和自谋职业。建设城乡统一的劳动力市场,促进城乡劳动者平等就业和自由流动。做好下岗失业和关闭破产企业人员再就业工作,帮助"零就业家庭"和就业困难人员就业,加强高校毕业生就业指导和服务,推进退役军人安置改革。发展和谐劳动关系,推行劳动合同制度,着力解决企业拖欠工资问题,努力提高低收入者的工资水平,切实保障劳动者合法权益。力争全市新增城镇就业人员7.5万人,实现下岗失业人员再就业2.5万人以上,其中就业困难对象0.9万人,基本消除"零就业家庭"。加强农村劳动力培训,提高农民科学种养技术水平和转移就业能力,培养造就新型农民。完成引导性培训6万人、技能性培训6万人,新增农村劳动力转移就业15万人,劳务总收入突破130亿元。把基本养老保险扩面、征缴、清欠工作列为各县市区年度考核目标,采取强力措施,确保各级各类用人单位依法参加基本养老保险,切实做到应保尽保、应收尽收,逐步实现基本养老保险全覆盖。完善企业职工基本养老保险市级统筹,健全城镇职工基本医疗保险和失业、工伤、生育保险制度。启动以大病统筹为主的城镇居民基本医疗保险试点。积极推行适合农民工特点的工伤、医疗、养老等社会保险制度,维护农民工的合法权益,让农民工政治上有地位、生活上有保障。健全城乡居民最低生活保障制度、城乡医疗救助制度、城市生活无着的流浪乞讨人员救助制度。高度关注弱势群体和困难群众生产生活问题,抓好城乡低保标准的落实,扎实做好城镇低保户、农村困难户救济救助工作。认真落实优抚政策,切实抓好防灾减灾救灾工作,支持慈善事业发展。新增城镇企业职工基本养老保险参保人数1.5万人,城镇职工基本医疗保险参保人数1.6万人;把农村低保人均月补差标准提高到30元、农村"五保"集中供养率提高到30%。

全面发展各项社会事业。坚持把教育放在优先发展的战略地位,加快各级各类教育发展,促进教育公平。完善农村义务教育经费保障机制,加大对义务教育的投入力度,优化学校布局,整合教育资源,加强教育设施配套建设,继续实施农村中小学校舍维修改造工程,促进义务教育均衡发展。积极创造条件,逐步实行以区为主的办学体制,理顺中心城区教育投入和管理体制。加快市二十一小、十七中、特殊教育学校建设,新开工建设2所学校,改建、扩建9所学校,缓解中心城区中小学入学压力。整合中等专业教育资源,大力发展中高等职业教育。提高高等教育质量,引导高校服务地方经济和社会发展。健全公共卫生和医疗服务体系,加强农村卫生工作,搞好城市社区卫生服务,致力解决群众看病难、看病贵问题。做好重大传染病防治工作,健全覆盖城乡的公共卫生和医疗服务体系。积极发展体育事业,开展"全民健身与奥运同行"主题活动,加快市体育中心配套建设。把稳定低生育水平作为人口和计划生育工作的首要任务,坚持利益导向与从严管理并重,完善机制,兑现奖惩,提高出生人口素质,统筹解决出生人口性别比偏高、人口老龄化、流动人口超生等问题。

切实做好安全生产和社会稳定工作。高度重视信访工作,按照分级负责、属地管理的原则,转变工作理念,畅通信访渠道,创新体制机制,落实信访工作责任制,依法维护信访秩序,引导群众合理表达诉求,及时解决群众信访反映的问题。深入开展矛盾纠纷排查调处工作,充分发挥复议、仲裁、民调在解决行政和民事纠纷方面的作用,从源头上化解矛盾。健全社会预警体系和突发公共事件应急机制,积极预防和妥善处置群体性事件,努力提高处置突发事件的能力。进一步加强社会治安综合治理,完善社会治安防控体系,大力开展平安创建活动,营造安居乐业的社会环境。认真落实国家、省、市出台的各项安全生产措施,严格落实安全生产责任制,加大专项治理和隐患整治力度,严防重特大安全事故发生,切实保障人民群众的生命财产安全。建立完善食品药品安全监管网络,整合监管力量,扎实开展专项整治,保障人民群众饮食用药安全。

加强精神文明和民主法制建设。全面加强社会公德、职业道德和家庭美德教育,提高全民思想道德素质。深入开展"文明城市"、"文明景区"创建活动和文明单位、文明行业、文明社区、文明村镇创建工作。切实搞好科技、文化、卫生"三下乡"和法律、科教、文体、卫生"四进社区"活动,加强农村文化阵地建设,进一步丰富群众文化生活。大力推进政务公开,确保权力公正透明运行。健全科学、民主决策机制,完善重大事项集体决策、专家咨询、社会公示与听证、决策评估等制度,推进决策的科学化、民主化。坚持依法治市,加强法制宣传教育,提高全社会的法制意识。加强城乡基层民主法制建设。自觉接受人大及其常委会的法律监督和政协的民主监督,认真办理人大代表建议和政协委员提案。广泛听取各民主党派、工商联和无党派人士的意见和建议。充分发挥工会、妇联、共青团和行业协会的作用。

加强国防动员和国防教育工作,关心支持驻宛解放军、武警部队建设和民兵预备役工作。积极开展"全国双拥模范城"创建活动,增强军政军民团结。认真做好民族宗教、侨务外事、统计、物价、气象、地震、史志、残疾人等工作。

折叠编辑本段自身建设

按照"为民、务实、高效、清廉"的要求,围绕建设行为规范、公正透明、勤政高效、清正廉洁政府的目标,积极推进政府自身改革与建设。

(一)转变政府职能,提高施政水平。按照精简、统一、效能的原则和决策、执行、监督相协调的要求,进一步深化行政管理体制改革,搞好经济调节和市场监管,强化社会管理和公共服务职能。继续深化行政审批制度改革,进一步清理、减少行政审批事项。加强行政效能监察,严格落实行政问责制,建立政府绩效评估制度,提高政府效能。

(二)坚持依法行政,规范行政行为。深入贯彻《行政许可法》和《全面推进依法行政实施纲要》,规范行政行为,提高依法行政水平。落实行政执法责任制、评议考核制、执法过错和行政违纪追究制,严厉查处违法违纪、失职、滥用职权、执法腐败等行为,切实做到有权必有责、用权受监督、侵权要赔偿、违法要追究。

(三)强化服务意识,优化发展环境。以创建服务型机关为载体,强化宗旨观念,提高服务水平。建立招商代办制,全面推行首问负责制、限期办结制和服务承诺制。规范完善行政服务中心建设。加快推进电子政务建设。改进完善行风评议。加大经济审计力度。集中开展涉企立项审批整治、涉企收费项目清理和涉企检查,严厉查处一批典型案例,致力改善投资经营环境。

(四)加强政风建设,狠抓工作落实。积极开展以"讲正气、树新风"为主题的机关思想作风教育整顿活动,切实解决机关干部作风中存在的"浮躁、懒散、官僚、争利、贪玩"五个方面的突出问题,严格落实《南阳市机关工作人员转变作风十禁止》规定,大力倡导八个方面的良好作风。坚持从严治政、廉洁从政,全面落实廉政建设责任制,深入开展廉政建设和反腐败斗争,努力从源头上预防和治理腐败。增强忧患意识、公仆意识、节俭意识,牢固树立过紧日子的思想,加强公共财政支出管理,规范政府机关公务接待,禁止公费旅游和非招商引资的外出考察。力争全年各行政事业单位办公经费节约10%以上。改进目标管理,强化政务督查,狠抓工作落实,确保圆满完成各项目标任务。
